# A Study of Sensory Nerve Conduction in Pre- and Post-immunoglobulin Treatment of Guillain-Barré Syndrome

**DOI:** 10.7759/cureus.51673

**Published:** 2024-01-04

**Authors:** Prashanth A, Vinod Shende, Sachin Pawar

**Affiliations:** 1 Physiology, Mahatma Gandhi Institute of Medical Sciences, Sevagram, IND

**Keywords:** snap, sensory nerve conduction, ivig, ncs, gbs

## Abstract

Background: Guillain-Barré syndrome (GBS) is a condition characterized by acute and progressive weakness that impacts the limbs, facial muscles, and bulbar muscles due to acute polyneuro-radiculopathy. Typically, an infection that results in immune-mediated nerve dysfunction is what starts the disease. Patients often encounter paresthesia or discomfort before progressing to muscle weakness, initially in the lower extremities (which may include some proximal components) and subsequently in the upper extremities. The features of polyneuropathy identified during electrophysiology tests, bolstered by evidence of acquired demyelination in the nerve conduction study (NCS), support the clinical diagnosis of GBS. In peripheral neuropathies, NCS often reveals abnormalities in nerve conduction parameters. A specific pattern observed in the sensory nerve conduction study (SNCS), referred to as "sural sparing," signifies that the sural nerve, located near the calf muscles, remains relatively unaffected compared to other sensory nerves. Very few studies have been conducted to investigate improvements in sensory nerve conduction (SNC) parameters before and after intravenous immunoglobulin (IVIG), offering limited clinical correlation for the recovery and prognosis of the disease. The study aimed to observe the NCS parameters of the sensory nerves in both the upper and lower limbs, before and after the infusion of IVIG.

Methodology: This study was an observational investigation conducted in the neurophysiology laboratory of the Physiology Department at a rural medical college in central India. Fifty clinically diagnosed cases of GBS aged between 18 and 60 years were referred from the Department of Medicine to the Physiology Department for conducting the NCS. Basic sociodemographic information, along with clinical history, was collected. Subsequently, the RMS EMG EP Mark-II machine was employed to examine the sensory nerve action potentials (SNAPs), such as amplitude (in mV) and conduction velocity (in ms), of the sensory nerves in both the upper and lower limbs before and after IVIG infusion. The IVIG infusion occurs within one week of clinically diagnosing GBS. Following an initial NCS, a second NCS follow-up study was conducted one week after the IVIG infusion to analyze the changing trend in sensory nerves.

Results: Upon analysis, no significant correlation was observed between the pre- and post-IVIG SNAPs of the median and ulnar nerves. However, the sural nerve conduction velocity's p-value of 0.033 demonstrated statistical significance, suggesting that the sural nerve is comparatively spared, confirming sural sparing. However, the SNAP of the sensory nerves in GBS patients showed a significant improvement overall, and only NCS quantified the percentage of improvement.

Conclusion: According to the study, the NCS of sensory nerves showed a positive change in the parameters examined before and after the infusion of IVIG. This underscores the timely intervention of GBS with IVIG, and conducting the sensory conduction study diligently will enhance knowledge about the recovery period. Additionally, it supports the treating physician in making informed interventions based on the results post-IVIG infusion. This enhancement in the sensory nerves can only be quantified through NCS.

## Introduction

Guillain-Barré syndrome (GBS), an uncommon but extremely dangerous immune-mediated polyneuro-radiculopathy, has intrigued medical professionals. It is described as the autoimmune degeneration of peripheral nervous system nerves, resulting in symptoms such as tingling, numbness, and weakening of the muscles, which can quickly progress to complete paralysis [1,2]. Sensory symptoms, such as tingling or numbness, typically initiate in the distant parts of the body and exhibit a symmetrical pattern. GBS encompasses two common subtypes, namely acute inflammatory demyelinating polyneuropathy (AIDP) and acute motor axonal neuropathy (AMAN). A rarer subtype, Miller Fisher syndrome (MFS), is distinguished by symptoms like ophthalmoplegia, ataxia, and areflexia. In general, GBS displays considerable variability in its clinical course, severity, and outcomes [3]. GBS, a heterogeneous disorder with a global incidence rate of one to two cases per 100,000 people, implies that the likelihood of an individual developing GBS in their lifetime is approximately one in 1000 [4,5]. The syndrome shows a gender predilection, affecting men approximately 1.5 times more frequently than women. Major predisposing causes include various infections, such as Campylobacter jejuni, cytomegalovirus, Epstein-Barr virus, and Mycoplasma pneumoniae, and GBS has been reported following influenza virus infection as well [6].

High-dose intravenous immunoglobulin (IVIG) and plasma exchange (PE) therapies have demonstrated effectiveness in alleviating the severity of symptoms associated with GBS. GBS is commonly associated with nonspecific infections or trigger events such as trauma, surgery, or immunization, which typically happen a few days to weeks before neurological symptoms. Younger patients are likely to have better prognosis and course of action. [7]. GBS, an autoimmune condition triggered by the body's defense mechanism reacting to an infectious pathogen containing a nerve-related antigen, has led to immune therapies as standard treatments, such as PE or IVIG. [8,9]. This potentially life-threatening condition poses challenges due to the variability in its clinical presentation and limits the understanding of its optimal timing of treatment initiation, especially concerning immunoglobulin therapy [10]. Investigating sensory nerve conduction (SNC) in the context of GBS is paramount, promising to provide comprehensive insights into the mechanisms, correlations, and timing of treatment. Immunoglobulin therapy, also known as IVIG, plays a pivotal role in this context, involving the intravenous administration of concentrated antibodies derived from human plasma to modulate the immune system's response. Its mechanisms encompass immunomodulation, anti-inflammatory effects, and pathogen neutralization, making it a valuable treatment for autoimmune diseases, immunodeficiency disorders, and certain infections [11]. While its benefits include disease management, potential drawbacks encompass adverse reactions, limited duration of effect, risk of infection transmission, and the possibility of allergic responses [12].

Therefore, the use of immunoglobulin therapy necessitates careful consideration, specific to the patient's condition and needs. In essence, the study of SNC in Pre- and post-immunoglobulin treatment of GBS is poised to address a notable gap in current GBS research. The essential need to address significant gaps in the current knowledge and investigate the nerve conduction parameters of GBS before and after the IVIG protocol motivated this research, particularly directed toward sensory nerves.

## Materials and methods

Study design and setting

This was an observational study prepared and reported using Strengthening the Reporting of Observational Studies in Epidemiology (STROBE) principles. The Institutional Ethics Committee reviewed and approved it with reference number MGIMS/IEC/PHY/92/2023. Over the 18-month duration, the study was carried out in the neurophysiology Laboratory of the Department of Physiology. Patients were referred from the Department of Medicine at a rural medical college in central India.

Study participants

Fifty diagnosed patients of the central Indian population with GBS were referred from the Department of Medicine to the Department of Physiology for the nerve conduction study (NCS). The patients were between the ages of 18 and 60 years. For the prevalence of 2%, with a confidence interval (CI) of 95% and design effect 1, we arrived at a minimum sample size of 31 [13]. A total of 50 participants were included, further categorized as pre-immunoglobulin therapy and post-immunoglobulin therapy. Written informed consent was obtained from all the patients in this study.

Selection criteria

Inclusion criteria included clinically diagnosed GBS patients of the age group between 18 and 60 years and of both genders who gave written informed consent. Patients with a recent history of fever, respiratory or gastrointestinal infection, surgery, and symptoms such as inability to walk for >10 minutes independently, rapid progression of weakness, severe autonomic or swallowing dysfunction, or respiratory insufficiency and their progression within one week from the time of diagnosis of GBS post-admission to the hospital were selected [13].

Exclusion criteria excluded patients with cardiac pacemakers or cardiac pathology, myelopathy, myopathy, neuromuscular junction disorders like myasthenia gravis, and those who did not give consent and were not willing to participate in the study.

Data sources and measurement of variables

Basic sociodemographic information was collected from each patient, along with clinical history, such as recent history of fever, surgery, weakness of limbs, and infection. The RMS EMG EP Mark-II machine used in this investigation was located in the neurophysiology laboratory of the Physiology Department at a rural medical college in central India. To reduce the number of errors, the same researcher conducted all of the tests at a constant room temperature (30°C) [14].

Electrophysiological evaluation of Guillain-Barré syndrome

A sensory nerve conduction study (SNCS) was done antidromically involving stimulation of sensory nerves proximally and recording sensory nerve action potentials (SNAPs) with electrodes placed distally over the dermatome distribution. The nerves tested were bilateral median, ulnar, and sural.

A pair of recording electrodes are placed in line over the nerve being studied, at an interelectrode distance of 2.5 cm to 4 cm, with the active electrode placed closest to the stimulator. For sensory nerve study, the stimulus strength duration of 100 μs, sweep speed of 2 ms/D, and filter between 20 Hz to 3 KHz and a current in the range of 5 mA to 30 mA to achieve supramaximal stimulation of the nerve was set [15].

SNCS was conducted on the bilateral median, ulnar, and sural nerves of GBS patients within one week from the time of diagnosis. The standard GBS therapy protocol was followed to treat the patients after the initial NCS, and a follow-up NCS was performed one week following the immunoglobulin infusion [16,17].

Statistical analysis

A standardized data collection form was used to abstract all of the information that was gathered. After the data was collected, it was tabulated, and SPSS software version 27 (SPSS Inc., Chicago, US) was used for the necessary statistical tests and statistical analysis. The values of the research parameters are shown as mean ± standard deviation (SD), and the means were compared using an unpaired Student's t-test. Statistics indicate that a P-value below 0.05 is considered statistically significant.

## Results

The research included 50 diagnosed GBS patients between the age group of 18 and 60 years, satisfying the inclusion criteria mentioned.

The mean and SD of right and left median nerve amplitudes at pre-treatment were 32.26±20.67 µV and 35.60±24.83 µV; at post-treatment, they were 37.39±17.26 µV and 34.92±19.41 µV, respectively. The mean scores of right and left median nerve conduction velocities at pre-treatment were 49.02±9.74 m/s and 49.05±10.36 m/s; at post-treatment, they were 52.08±10.19 m/s and 48.88±9.20 m/s, respectively. The corresponding t-values of bilateral median amplitudes were 1.03 and 0.11; conduction velocities were 1.13 and 0.10. The bilateral median nerve amplitudes have a probability value (p-value) of 0.310 and 0.908 and conduction velocities of 0.265 and 0.917, respectively. By using Student’s paired t-test, statistically no significant difference was found, as depicted in Table 1.

**Table 1:** Comparison of the median nerve (sensory) parameters at pre- and post-treatment R, right; L, left; Amp, amplitude; CV, conduction velocity; mv, microvolt; m/s, meter per second; IVIG, intravenous immunoglobulin

Median nerve	Pre-IVIG	Post-IVIG	Mean difference	t-value	p-value
R median (Amp) mv	32.26±20.67	37.39±17.26	5.13±26.70	1.03	0.310 (NS)
R median (CV) m/s	49.02±9.74	52.08±10.19	3.05±14.48	1.13	0.265 (NS)
L median (Amp) mv	35.60±24.83	34.92±19.41	0.68±30.32	0.11	0.908 (NS)
L median (CV) m/s	49.05±10.36	48.88±9.20	0.17±8.64	0.10	0.917 (NS)

Following the treatment, bilateral median SNAPs, encompassing amplitude and conduction velocity, exhibit significant improvement. This finding suggests a notable recovery in sensory nerve function, as delineated in Table 2.

**Table 2:** The total percentage of improvement in the bilateral median nerve (sensory) post-treatment

Median nerve	Percentage of improvement after treatment
Amplitude	65%
Conduction velocity	59%

The mean and SD of right and left ulnar nerve amplitudes at pre-treatment were 40.67±25.36 µV and 30.92±23.25 µV; at post-treatment, they were 39.84±23.53 µV and 49.84±7.55 µV, respectively. The mean scores of right and left ulnar nerve conduction velocities at pre-treatment were 55.77±17.53 m/s and 49.22±12.05 m/s; at post-treatment, they were 49.84±7.55 m/s and 48.64±7.99 m/s, respectively. The corresponding t-values of bilateral ulnar amplitudes were 0.11 and 0.73; conduction velocities were 1.62 and 0.25. The bilateral ulnar nerve amplitudes have a probability value (p-value) of 0.90 and 0.47 and conduction velocities of 0.11 and 0.80, respectively. By using Student’s paired t-test, statistically no significant difference was found, as depicted in Table 3. 

**Table 3:** Comparison of the ulnar nerve (Sensory) score at pre- and post-treatment R, right; L, left; Amp, amplitude; CV, conduction velocity; mv, microvolt; m/s, meter per second; IVIG, intravenous immunoglobulin

Ulnar nerve	Pre-IVIG	Post-IVIG	Mean difference	t-value	p-value
R ulnar (Amp) mv	40.67±25.36	39.84±23.53	0.82±37.37	0.11	0.90 (NS)
R ulnar (CV) m/s	55.77±17.53	49.84±7.55	5.92±18.94	1.62	0.11 (NS)
L ulnar (Amp) mv	30.92±23.25	35.71±25.40	4.78±33.43	0.73	0.47 (NS)
L ulnar (CV) m/s	49.22±12.05	48.64±7.99	0.58±11.74	0.25	0.80 (NS)

Following the treatment, bilateral ulnar nerve SNAPs, encompassing amplitude and conduction velocity, exhibit significant improvement. This finding suggests a notable recovery in sensory nerve function, as delineated in Table 4.

**Table 4:** The total percentage of improvement in the bilateral ulnar nerve (sensory) post-treatment

Ulnar nerve	Percentage of improvement after treatment
Amplitude	57%
Conduction velocity	52&

The mean and SD of right and left sural nerve amplitudes at pre-treatment were 37.31±20.66 µV and 40.94±22.26 µV; at post-treatment, they were 41±20.83 µV and 40.89±29.79 µV, respectively. The mean scores of right and left sural nerve conduction velocities at pre-treatment were 53.58±11.18 m/s and 51.95±14.73 m/s; at post-treatment, they were 58.75±13.79 m/s and 54.27±8.35 m/s, respectively. The corresponding t values of bilateral Sural amplitudes were 0.70 and 0.009; conduction velocities were 2.24 and 0.84. The bilateral ulnar nerve amplitudes have a probability value (p-value) of 0.48 and 0.99 and conduction velocities of 0.033 and 0.40, respectively. By using Student’s paired t-test, right sural nerve conduction velocity shows statistical significance with the p-value of <0.05, as depicted in Table 3. 

**Table 5:** Comparison of the sural nerve parameters at pre- and post-treatment R, right; L, left; Amp, amplitude; CV, conduction velocity; mv, microvolt; m/s, meter per second; IVIG, intravenous immunoglobulin

Sural nerve	Pre-IVIG	Post-IVIG	Mean difference	t-value	p-value
R sural (Amp) mv	37.31±20.66	41±20.83	3.69±28.86	0.70	0.48 (NS)
R sural (CV) m/s	53.58±11.18	58.75±13.79	5.17±12.64	2.24	0.033 (S)
L sural (Amp) mv	40.94±22.26	40.89±29.79	0.05±33.04	0.009	0.99 (NS)
L sural (CV) m/s	51.95±14.73	54.27±8.35	2.32±15.11	0.84	0.40 (NS)

Following the treatment, bilateral sural nerve SNAPs, encompassing amplitude and conduction velocity, exhibit significant improvement. This finding suggests a notable recovery in sensory nerve function, as delineated in Table 6.

**Table 6:** The total percentage of improvement in the bilateral sural nerve post-treatment

Sural nerve	Percentage of improvement after treatment
Amplitude	53%
Conduction velocity	60%

**Figure 1:**
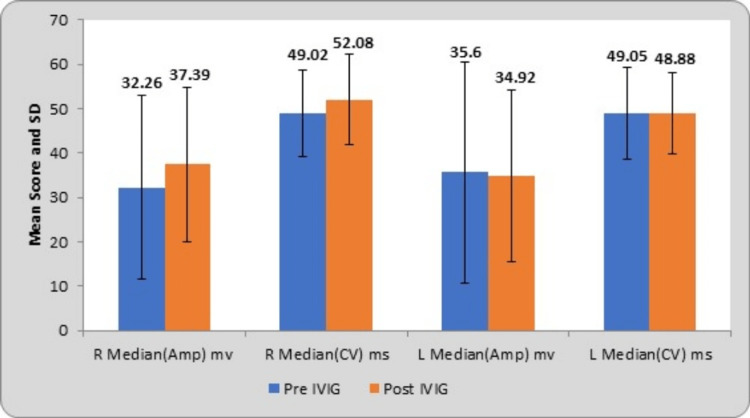
Comparison of the median nerve (sensory) parameters at pre- and post-IVIG treatment R, right; L, left; Amp, amplitude; CV, conduction velocity; mv, microvolt; m/s, meter per second; IVIG, intravenous immunoglobulin; SD, standard deviation

**Figure 2:**
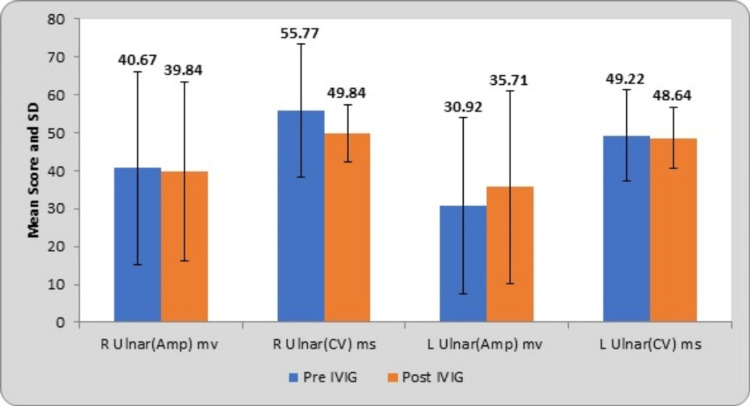
Comparison of the ulnar nerve (sensory) parameters at pre- and post-treatment R, right; L, left; Amp, amplitude; CV, conduction velocity; mv, microvolt; m/s, meter per second; IVIG, intravenous immunoglobulin; SD, standard deviation

**Figure 3:**
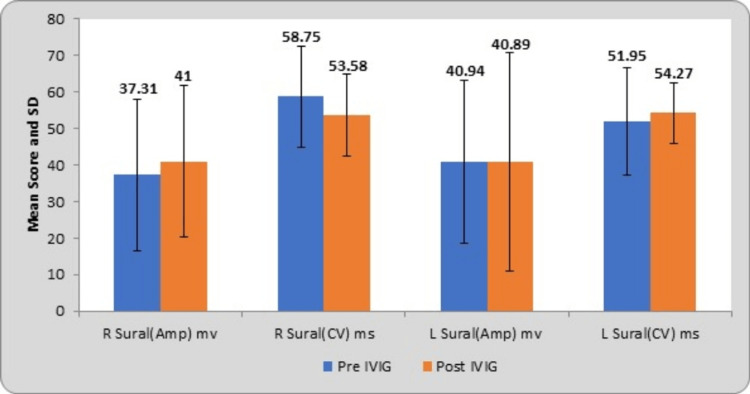
Comparison of the sural nerve parameters at pre- and post-treatment R, right; L, left; Amp, amplitude; CV, conduction velocity; mv, microvolt; m/s, meter per second; IVIG, intravenous immunoglobulin; SD, standard deviation

Graphical representation of the sensory nerves such as median, ulnar, and sural nerves with their NCS parameters is presented in Figures 1, 2, and 3. 

## Discussion

According to the literature search, no specific original research study has been identified that elucidates the electrophysiological changes pertaining to sensory nerves occurring in GBS patients before and after intravenous infusion of immunoglobulin. This absence of research impedes the depiction of alterations in sensory nerve function, hindering a comprehensive understanding of the clinical correlation of the disease.

GBS is broadly classified as demyelinating and axonal subtypes based on electrophysiological and histopathological observations. NCS continues to be the cornerstone in electrodiagnosis and the categorization of subtypes. AIDP is characterized by conduction slowing, conduction block, and temporal dispersion. Axonal neuropathy, on the other hand, lacks demyelinating features and exhibits a decrease in distal compound muscle action potentials (CMAPs) and SNAPs. In nearly all cases of AIDP, there is impairment in SNC, particularly in the distal nerve segment, while the sural response remains normal or relatively spared [18].

Regardless of the criteria employed for electrophysiological subtyping, the occurrence of abnormalities in median and ulnar sensory potentials, as well as the sural sparing pattern, was notably more frequent in demyelinating subtypes compared to all other subtypes. Nagappa et al. also noted that variations in abnormalities observed in sensory conduction studies are common and contingent on the duration of the disease. The presence or absence of sensory nerve involvement may indicate different underlying immunopathogenic mechanisms [19].

The possibility of reversible conduction failure, similar to what is observed in motor nerves, is suggested by consecutive examinations of sensory potentials. These studies reveal dynamic changes analogous to those observed in motor nerves. Even in cases where baseline sensory conductions appeared normal, sequential recordings of sensory conductions uncovered that a majority of GBS patients exhibited sub-clinically affected sensory fibers [20]. As per Capasso et al, significant increases in SNAP amplitudes were noted in 34% of initially normal nerves. This implies that some sensory fibers were impacted at baseline, although not to an extent that caused SNAP to fall below the normal limit. This aligns with our findings indicating that amplitudes were initially relatively affected before the infusion of IVIG and subsequently improved after the IVIG infusion. Nagasawa et al. reported that sensory conduction abnormalities were noted exclusively in demyelinating group [21]

Jin et al.'s research indicates that, in comparison to the median, ulnar, or radial nerves, the sensory potentials of the sural nerve are relatively preserved. [22]. An informative electrodiagnostic indicator for inflammatory demyelinating neuropathies, including GBS, is the phenomenon termed "sural sparing" [23]. Consistent with this observation, our research reveals a noteworthy enhancement in conduction velocity in the sural nerve, indicating a pattern consistent with sural sparing and the sural nerve is preserved during such diseases. In a multi-center retrospective study, the identification of sural sparing demonstrated a specificity of 0.95, a sensitivity of 0.41, and a positive predictive value of 8.20 when diagnosing early demyelinating GBS [24].

As per our study, when clinical intervention occurred within the first week of the onset of GBS symptoms, post-immunoglobulin infusion, there was a significant improvement of 65%, 57%, and 53% in the amplitude of bilateral median, ulnar, and sural sensory nerves, respectively. Additionally, notable improvements of 59%, 52%, and 60% were observed in the conduction velocity of bilateral median, ulnar, and sural sensory nerves, respectively, signifying a recovery in sensory nerve function. In a follow-up study conducted after two weeks, Kuwabara et al. observed that the electrophysiological changes had returned to normal [25]. It is expected that improvements in NCS parameters correlate with clinical improvement after IVIG.

In general, the clinical outcome of any GBS patient is likely to be correlated with the results of electrophysiological nerve conduction studies conducted before and after IVIG treatment. This approach provides valuable insights into the percentage of involvement in sensory nerves, allowing clinicians to make informed treatment decisions for the patient. Timely diagnosis and prompt initiation of treatment have the potential to enhance the prognosis for all subtypes of GBS.

Limitations

Like any research project, this one had its share of constraints. The GBS patients were not classified into subtypes. The sample size is significantly less, and a larger sample size with serial nerve conduction will provide a more accurate estimate of the percentage of sensory nerves involved. The study did not cover the pediatric age group; however, this can be added in future studies to improve results across a wider age range. Furthermore, although it was not used in the study, a different statistical method might be applied to assess a composite score of several indications, which could advance the field.

## Conclusions

The study emphasizes that the clinical outcome of GBS patients is strongly supported by the findings of the electrophysiological study. Specifically, the treatment protocol involving IVIG initiation within the first week of symptom onset shows evidence of reversible conduction in SNAPs. This is evident from the improvement observed in the potentials of our sensory nerves. In essence, the findings underscore the crucial role of timely diagnosis and the prompt initiation of treatment in enhancing the prognosis for individuals with all subtypes of GBS.

## References

[1] Walling AD, Dickson G (2013). Guillain-Barré syndrome. Am Fam Physician.

[2] Hughes RA, Cornblath DR (2005). Guillain-Barré syndrome. Lancet.

[3] Hadden RD, Cornblath DR, Hughes RA, Zielasek J, Hartung HP, Toyka KV, Swan AV (1998). Electrophysiological classification of Guillain-Barré syndrome: clinical associations and outcome. Ann Neurol.

[4] Pithadia AB, Kakadia N (2010). Guillain-Barré syndrome (GBS). Pharmacol Rep.

[5] Chiò A, Cocito D, Leone M, Giordana MT, Mora G, Mutani R (2003). Guillain-Barré syndrome: a prospective, population-based incidence and outcome survey. Neurology.

[6] Cosi V, Versino M (2006). Guillain-Barré syndrome. Neurol Sci.

[7] Feasby TE, Gilbert JJ, Brown WF, Bolton CF, Hahn AF, Koopman WF, Zochodne DW (1986). An acute axonal form of Guillain-Barré polyneuropathy. Brain.

[8] Sharma CM, Pandey RK, Kumawat BL, Khandelwal D, Acharya M (2016). Guillain-Barré syndrome in north-western India: demographic, clinical, electrophysiological profile, and assessment of prognostic factors. Ind J Med Spec.

[9] Korinthenberg R, Trollmann R, Felderhoff-Müser U (2020). Diagnosis and treatment of Guillain-Barré Syndrome in childhood and adolescence: an evidence- and consensus-based guideline. Eur J Paediatr Neurol.

[10] Chevret S, Hughes RA, Annane D (2017). Plasma exchange for Guillain-Barré syndrome. Cochrane Database Syst Rev.

[11] Verboon C, Harbo T, Cornblath DR (2021). Intravenous immunoglobulin treatment for mild Guillain-Barré syndrome: an international observational study. J Neurol Neurosurg Psychiatry.

[12] Guo Y, Tian X, Wang X, Xiao Z (2018). Adverse effects of immunoglobulin therapy. Front Immunol.

[13] Leonhard SE, Mandarakas MR, Gondim FA (2019). Diagnosis and management of Guillain-Barré syndrome in ten steps. Nat Rev Neurol.

[14] Halar EM, DeLisa JA, Soine TL (1983). Nerve conduction studies in upper extremities: skin temperature corrections. Arch Phys Med Rehabil.

[15] Pawar SM, Taksande AB, Singh R (2012). Effect of body mass index on parameters of nerve conduction study in Indian population. Indian J Physiol Pharmacol.

[16] Nguyen TP, Taylor RS (2023). Guillain-Barre syndrome. https://www.ncbi.nlm.nih.gov/books/NBK532254/.

[17] van Doorn PA, Ruts L, Jacobs BC (2008). Clinical features, pathogenesis, and treatment of Guillain-Barré syndrome. Lancet Neurol.

[18] Huynh R, Cabrero FR (2023). Electrodiagnostic evaluation of acute inflammatory demyelinating polyneuropathy. https://www.ncbi.nlm.nih.gov/books/NBK562217/.

[19] Nagappa M, Wahatule R, Bindu PS, Sinha S, Taly AB (2022). Spectrum of sensory conduction abnormalities in Guillain Barre syndrome. Neurol India.

[20] Capasso M, Notturno F, Manzoli C, Uncini A (2011). Involvement of sensory fibres in axonal subtypes of Guillain-Barre syndrome. J Neurol Neurosurg Psychiatry.

[21] Nagasawa K, Kuwabara S, Misawa S (2006). Electrophysiological subtypes and prognosis of childhood Guillain-Barré syndrome in Japan. Muscle Nerve.

[22] Jin J, Hu F, Qin X, Liu X, Li M, Dang Y, Dang J (2018). Very early neurophysiological study in Guillain-Barre syndrome. Eur Neurol.

[23] Bromberg MB, Albers JW (1993). Patterns of sensory nerve conduction abnormalities in demyelinating and axonal peripheral nerve disorders. Muscle Nerve.

[24] Derksen A, Ritter C, Athar P (2014). Sural sparing pattern discriminates Guillain-Barré syndrome from its mimics. Muscle Nerve.

[25] Kuwabara S, Ogawara K, Misawa S (2004). Does Campylobacter jejuni infection elicit "demyelinating" Guillain-Barre syndrome?. Neurology.

